# Supplementary material for: The role of metacognition in promoting deep learning in MOOCs during COVID-19 pandemic
Source: PeerJ Comput Sci. 2022 Jun 15;8:e945. doi: 10.7717/peerj-cs.945 (PMC9299274; doi:10.7717/peerj-cs.945)
Supplement: Supplemental Information 2 [file peerj-cs-08-945-s002.docx]

بطاقة تقييم التعلم العميق

| م | جوانب البطاقة | لا أوافق بشدة | لا أوافق | محايد | أوافق | أوافق بشدة |
| --- | --- | --- | --- | --- | --- | --- |
| التفكير النقدي | | | | | | |
| 1 | اختبار تأثير المتغير المستقل على المتغير التابع. |  |  |  |  |  |
| 2 | تحديد أسئلة الدراسة للإجابة عليها. |  |  |  |  |  |
| 3 | صياغة إجابات محتملة يمكن اختبارها لكل سؤال. |  |  |  |  |  |
| 4 | كتابة فرضية صفرية وأخرى بديلة للدراسة. |  |  |  |  |  |
| 5 | التمييز بين الفرضيات التي يمكن اختبارها وصفيًا والتي يمكن اختبارها كميًا. |  |  |  |  |  |
| 6 | التوصل للنتائج الختامية في وقت قصير. |  |  |  |  |  |
| 7 | تضمين نتيجة دراسة سابقة في الدراسة المختارة. |  |  |  |  |  |
| ربط المفاهيم (ربط المعرفة الجديدة بالحالية) | | | | | | |
| 8 | وضع معايير صياغة عنوان البحث الجيد في الاعتبار. |  |  |  |  |  |
| 9 | كتابة سؤال رئيس تجيب عنه الدراسة. |  |  |  |  |  |
| 10 | تحديد مجتمع الدراسة. |  |  |  |  |  |
| 11 | توثيق المراجع والمصادر. |  |  |  |  |  |
| 12 | تحديد المتغيرات المستقلة والتابعة والدخيلة. |  |  |  |  |  |
| 13 | وصف أسلوب تحديد العينة ونوعها. |  |  |  |  |  |
| 14 | تحديد القوانين والمبادئ والنظريات ذات الصلة بالموضوع. |  |  |  |  |  |
| تكوين مفاهيم جديدة | | | | | | |
| 15 | صياغة المصطلحات المرتبطة بالنتائج وأسبابها أو ظواهرها وشروطها. |  |  |  |  |  |
| 16 | وصف التصميم التجريبي المقترح. |  |  |  |  |  |
| 17 | التعرف على تقنيات وأدوات جمع البيانات. |  |  |  |  |  |
| 18 | تحديد البيانات أو النتائج ذات الصلة بالموضوع. |  |  |  |  |  |
| 19 | معالجة البيانات أو النتائج. |  |  |  |  |  |
