# Supplementary material for: The role of metacognition in promoting deep learning in MOOCs during COVID-19 pandemic
Source: PeerJ Comput Sci. 2022 Jun 15;8:e945. doi: 10.7717/peerj-cs.945 (PMC9299274; doi:10.7717/peerj-cs.945)
Supplement: Appendix S1 [file peerj-cs-08-945-s003.docx]

**Appendix**

**The assessment card of deep learning**

| Card aspects | | Strongly disagree | Disagree | Neither agree nor disagree | Agree | Strongly agree |
| --- | --- | --- | --- | --- | --- | --- |
| Critical thinking | | | | | | |
| 1 | Testing the effect of independent variable on the dependent one. |  |  |  |  |  |
| 2 | Identifying study questions to be answered. |  |  |  |  |  |
| 3 | Formulating probable answers that can be tested for every question. |  |  |  |  |  |
| 4 | Writing study null and alternative hypotheses. |  |  |  |  |  |
| 5 | Distinguishing between hypotheses that can be tested descriptively or quantitatively. |  |  |  |  |  |
| 6 | Concluding results in a shorter time. |  |  |  |  |  |
| 7 | Including the answer of a previous study in the topic of the chosen study. |  |  |  |  |  |
| Connecting concepts (connect new knowledge with what already knew) | | | | | | |
| 8 | Considering the criteria for formulating a good research title. |  |  |  |  |  |
| 9 | Writing a key question that the study will answer. |  |  |  |  |  |
| 10 | Identifying the study population. |  |  |  |  |  |
| 11 | Documenting references and resources. |  |  |  |  |  |
| 12 | Identifying independent, dependent and persistent variables. |  |  |  |  |  |
| 13 | Describing the sampling technique and type. |  |  |  |  |  |
| 14 | Identifying topic related laws, principles or theories. |  |  |  |  |  |
| Creating new concepts | | | | | | |
| 15 | Formulating the terms associated with the result and its causes or phenomena and their conditions. |  |  |  |  |  |
| 16 | Describing the proposed experimental design. |  |  |  |  |  |
| 17 | Identifying the data collection techniques and tools. |  |  |  |  |  |
| 18 | Identifying topic related data or results. |  |  |  |  |  |
| 19 | Processing data or results. |  |  |  |  |  |
